# Multimodal Counseling Interventions: Effect on Human Papilloma Virus Vaccination Acceptance

**DOI:** 10.3390/healthcare5040086

**Published:** 2017-11-06

**Authors:** Oroma Nwanodi, Helen Salisbury, Curtis Bay

**Affiliations:** 1 Obstetrics and Gynecology Locum Tenens, Salinas, CA 93902, USA; 2 College of Graduate Health Studies, A. T. Still University, Mesa, AZ 85206, USA; hsalisbury@atsu.edu; 3 Department of Interdisciplinary Sciences, A. T. Still University, Mesa, AZ 85026, USA; cbay@atsu.edu

**Keywords:** cervical cancer, counseling, human papilloma virus (HPV), HPV counseling, HPV-attributable diseases, HPV knowledge, HPV prophylaxis, HPV vaccination, HPV vaccination acceptance

## Abstract

Human papilloma virus (HPV) vaccine was developed to reduce HPV-attributable cancers, external genital warts (EGW), and recurrent respiratory papillomatosis. Adolescent HPV vaccination series completion rates are less than 40% in the United States of America, but up to 80% in Australia and the United Kingdom. Population-based herd immunity requires 80% or greater vaccination series completion rates. Pro-vaccination counseling facilitates increased vaccination rates. Multimodal counseling interventions may increase HPV vaccination series non-completers’ HPV-attributable disease knowledge and HPV-attributable disease prophylaxis (vaccination) acceptance over a brief 14-sentence counseling intervention. An online, 4-group, randomized controlled trial, with 260 or more participants per group, found that parents were more likely to accept HPV vaccination offers for their children than were childless young adults for themselves (68.2% and 52.9%). A combined audiovisual and patient health education handout (PHEH) intervention raised knowledge of HPV vaccination purpose, *p* = 0.02, and HPV vaccination acceptance for seven items, *p* < 0.001 to *p* = 0.023. The audiovisual intervention increased HPV vaccination acceptance for five items, *p* < 0.001 to *p* = 0.006. That HPV causes EGW, and that HPV vaccination prevents HPV-attributable diseases were better conveyed by the combined audiovisual and PHEH than the control 14-sentence counseling intervention alone.

## 1. Introduction

In the United States (U.S.), approximately 26,900 cases of high-risk human papilloma virus (HPV)-attributed genital and oropharyngeal cancers occur annually [1]. Overall, there are 38,793 HPV-attributed cancers annually in the U.S., an incidence of 11.7 per 100,000 persons [2]. High-risk oncogenic HPV types 16, 18, 31, 33, 45, 52, and 58 are associated with 92% of HPV-attributable cancers [2]. Low-risk non-oncogenic HPV types 6 and 11 are responsible for 96–100% of external genital warts (EGW) that have an incidence of 205 persons per 100,000 persons, affecting up to one million Americans annually [3,4,5]. Low-risk, non-oncogenic HPV Types 6 and 11 also cause 0.43 to 4.3 cases of recurrent respiratory papillomatosis (RRP) per 100,000 children, and 1.8 cases of RRP per 100,000 adults [6,7]. The prevalence of high-risk oncogenic HPV ranges from 16.6 to 65.3%, in American men ages 18 to 44 and American women ages 18 to 35, while low-risk non-oncogenic HPV prevalence ranges from 13.5 to 25.3% [3,8]. All told, this amounts to 75% of Americans experiencing an HPV infection once in their life [9].

The U.S. spent at least $700 million in 2009 on HPV-attributable diseases [10]. Global approval of HPV vaccines began in 2006. The quadrivalent and nonavalent HPV vaccines (4vHPV and 9vHPV) prevent HPV-attributed cervical cancer, EGW, and RRP [4,7,11]. 4vHPV provides immunity from HPV-6, -11, -16, and -18. 9vHPV also provides immunity from HPV-31, -33, -45, -52, and -58 [2].

In 2009, a school-based 65% 3-dose HPV vaccination rate for Australian females ages 12–26, reduced the incidence of female and male EGW by 16.7 and 20 percentage-points respectively [12]. Subsequently, Australia achieved a 79% vaccination rate [13,14,15]. Higher school-based 3-dose HPV vaccination rates are 85% in Brazil and 81% in Britain [14,15]. In pre-HPV vaccination approval China, parental HPV vaccine acceptance was 36.2% [16]. France, like the U.S. lacks a national HPV vaccination program [13]. France had a 38% adolescent female HPV vaccination series completion rate in 2013 [13]. This is closer to the U.S.’ primarily clinic-based 32% female 3-dose HPV vaccination rate, than that obtained by school-based vaccination programs worldwide [17]. Adolescent girls (13–17 years old) in the U.S. fared slightly better with a 40% vaccination rate in 2014 [9,18]. Conversely, adolescent boys in the U.S. fared worse with a 22% vaccination rate [9,18,19]. Underlying the importance of high HPV series vaccination rates, states with 3-dose adolescent female HPV vaccination rates of less than 33.9% in 2013 are associated with moderate and high age-adjusted cervical cancer incidence, 6.76 to 9.75 per 100,000 [20].

Parental HPV-vaccination acceptance may be most strongly associated with attitude, habit, intention, and subjective norms, providing direction for vaccination education [21]. Media campaigns targeted to the Latino population led to a 15% increase in immunizations in California [22]. A Vietnamese language media-led information and education campaign in Texas achieved a 21.5 percentage-point increased awareness of Hepatitis B, a 31.9 percentage-point increase in awareness of free childhood vaccinations, and a 14 percentage-point increase in awareness that Hepatitis B is sexually transmitted [23]. The Guildford County North Carolina HPV Campaign presentation to healthcare staff, parents, and school staff increased parents’ post-intervention knowledge of HPV-attributable disease by 31% [24]. After the presentation, 97% of Guilford County, North Carolina parents were supportive of a school-based HPV vaccination clinic [24]. Given that electronic-based HPV vaccine education can be 23% more costly than print-based HPV vaccine education, educational method relative effectiveness is important [25].

Public education can address adverse events of vaccines in general, the HPV vaccine specifically, and refute the assumption that HPV vaccination results in rebound increased sexual activity [26]. Public education can also address the epidemiology of HPV-attributed infections, susceptibility to HPV-attributed infections, and societal burden thereof, leading to HPV vaccination recommendation. As 19% of parents are unsupportive of HPV vaccination, unless all other eligible children receive HPV vaccination, the U.S. cannot achieve an 80% or greater HPV vaccination completion rate for grade-school age children, necessary for herd immunity [9,27]. Attainment of HPV herd immunity protecting the un-vaccinated would reduce HPV-attributed disease in the U.S. If counseling interventions can steer parents who initially would not accept HPV vaccination to accept HPV vaccination, an 80% or greater HPV vaccination completion rate is achievable. The objective of this quantitative comparative study was to evaluate whether multimodal counseling interventions increased HPV vaccination series non-completers’ knowledge of HPV-attributable disease and acceptance of HPV-attributable disease prophylaxis (vaccination), over a control 14-sentence counseling intervention.

## 2. Materials and Methods

**Figure 1:**
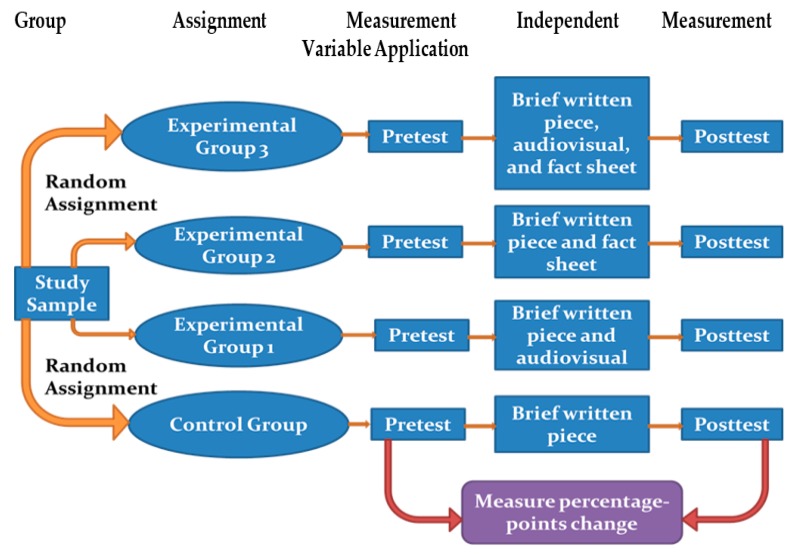
Study design flowchart. A single-blind, quantitative, four group, simple randomized, repeated measures, pre-test/post-test design with sample of young adults and parents from total survey respondents.

**Figure 2:**
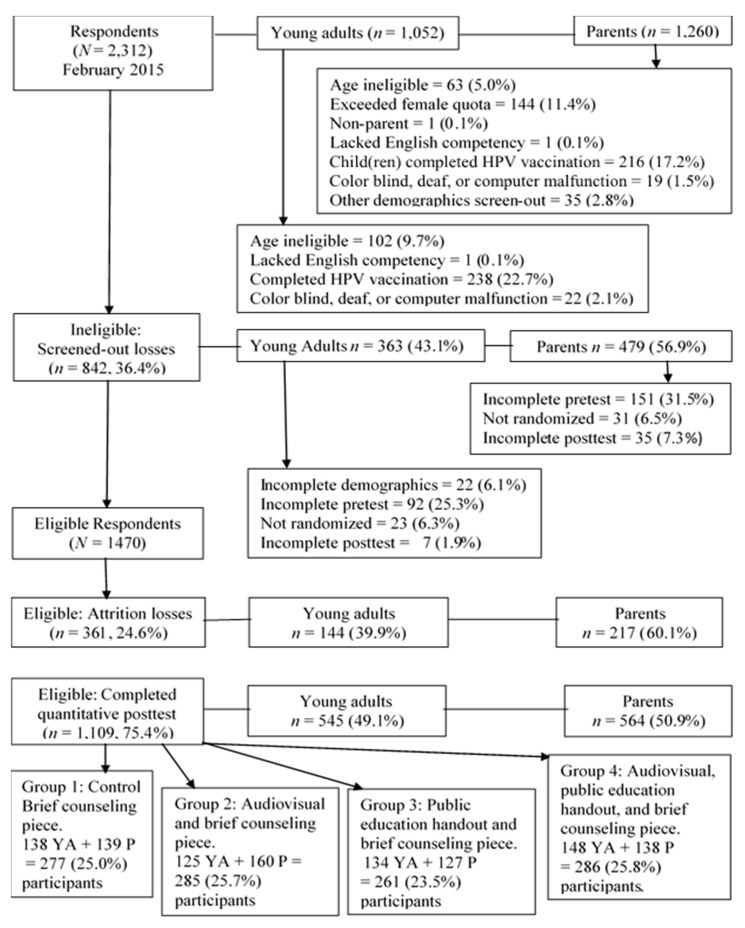
Participant flowchart. Sample of young adults and parents from total survey respondents. There were at least 260 participants per study group, comprised half of young adults and half of parents, with an approximately 60:40 female to male ratio, and a final sample size of 1109 participants.

We performed a single-blind, quantitative, four group, probability sampling, simple randomized, pre-test/post-test online survey (Figure 1) with a minimum of 260 participants per study group, comprised half of young adults and half of parents, about 60:40 female to male ratio, totaling 1109 participants overall (Figure 1 and Figure 2, and Table 1). Response tracking for partial and complete respondents was used. Invitation tracking for unopened or bounced (undeliverable) online invitation accounting was not used. All participants completed an online 25-item demographic questionnaire including initial screening items (Questionnaire S1–S4). All four groups responded to the pre-test 49-item HPV Knowledge and Acceptance survey, completed their assigned independent variable, and completed the post-test 49-item HPV Knowledge and Acceptance survey. All questionnaires had a young adult version and a parent version (Questionnaires S2 and S3). Quantitative analysis of survey items 1–25, from pre- and post-tests of all four groups, formed this study. Prior to data collection, this study received an exempt status from the A. T. Still University Institutional Review Board (Permission S1).

The first and control level counseling intervention received by all study groups was a 14-sentence informational brief, provided as Intervention S1 [28]. The second level counseling intervention comprised the 14-sentence informational brief and a 4.34-min audiovisual *Why vaccinate against HPV* [29]. The third level counseling intervention was the 14-sentence informational brief and a public health education handout (PHEH) based on the *Public Health Fact Sheet*: *Patient information about HPV and the HPV vaccine* (Intervention S2) [30]. The fourth level counseling intervention comprised the 14-sentence informational brief, the audiovisual, and the PHEH. Consents were obtained for all independent variable use (Permissions S2–S4). The HPV Knowledge and HPV Vaccination Acceptance survey, comprised of the Parental HPV Survey, validated for use with 5-point Likert scale coded responses with Cronbach’s alphas >0.95, a validated 3-item HPV acceptance questionnaire, both available without express consent for educational purposes, and a consented adaptation of focused interview questions [26,27,28].

In February 2015, SurveyMonkey Audience invited Americans 19 years and older to participate via age-based probability sampling with simple randomization to groups. SurveyMonkey Audience protocol determined the participation incentive. Eligible participants were age 19 or older, able to read and accept an online Survey Participation Consent in English, able to complete the online survey in English, and had not completed the HPV vaccination series. Young adults, age 19–26 years could participate offering opinions for themselves without regard to any child(ren) they may have. Young adults could not also participate as parents. Interested persons progressed to the inclusion/exclusion criteria items. Consent was implied once a potential participant proceeded to the survey instrument screen. Of 2312 initial respondents, 1470 were eligible, and 1109 eligible participants (75.4%) completed the survey (Figure 2).

The online survey host, SurveyMonkey Audience, directly invited potential participants from its database and maintained the anonymity of potential and actual study participants. Data lacking identifiers, IP, or email address tracking was collected via encrypted secure sockets layer/transport layer security technology (SSL/TLS) connections in February 2015. The survey lacked name, address, or contact information fields. Participant data storage followed the standard SurveyMonkey Audience anonymous data storage protocol: user authentication and user passwords for data access, and continued data encryption while stored in an audited secure data center. 

Statistical analysis was performed using IBM SPSS Statistics (IBM Corp., Armonk, NY, USA), package Version 22. Normality testing with Kolmogorov-Smirnov and Shapiro-Wilk tests at *p* < 0.05 ascertained a lack of normally distributed demographics, knowledge, and acceptance items responses. Therefore, count and frequency descriptive statistics were used for the sample description. Chi-square was used to ascertain randomization validity. Spearman’s rank correlation coefficient (ρ) was used for comparisons between interval and ordinal variables [31].

Quantitative HPV knowledge and HPV vaccination acceptance items were analyzed for the 1109 respondents who completed the survey through the end of the quantitative post-test. Responses were extracted and analyzed as two groups, as well as individually. Given the lack of normality and the use of four study groups, two-tailed non-parametric tests at alpha level α ≤ 0.05 were employed: Kruskal-Wallis analysis of variance by ranks (*χ*^2^), Mann-Whitney *U* test with Bonferroni correction, and Wilcoxon signed-ranks test (*T*).

## 3. Results

Childless young adults who had not completed a HPV vaccination series and parents whose child(ren) had not completed a HPV vaccination series were the study population. Prior completion of the HPV vaccination series was the primary cause of study ineligibility (Figure 2). The study had a 75.4% completion rate. Pre-test attrition was the most common form of attrition loss (67.3%). Table 1 and Figures S1–S4 describe the 1109 respondents who completed the survey through the end of the quantitative post-test. The resulting sample had a positively skewed age distribution, comprised of 545 childless young adults ages 19–26, and 564 parents age 27 years or older (Figure 2, Figures S1–S4 and Table 1). Childless young adults were more likely to have never married than were parents (64.8% and 9.4% respectively), and more likely to have had some college education than parents (35% and 26.2% respectively). However, parents were more likely to have graduated from college or graduate school than were childless young adults (41.1% and 16.5% versus 26.4% and 5.5%, respectively). Parents were more likely to accept offers of HPV vaccination for a child than were childless young adults for themselves (68.2% versus 52.9%, respectively). 

Chi-square analysis ascertained valid random assignment of participants to intervention groups based on 15 nominal independent variables excluding insurance status, marital status, race and ethnicity, and religion (Table 1). Human Papilloma Virus vaccination acceptance and insurance type were imbalanced (*p* = 0.029 and *p* = 0.038 respectively) (Table 1). Due to category assignments of less than five per intervention group, 2-sided Fisher’s exact tests were calculated for marital status and religion, resulting in *p* < 0.001 in each case. For race and ethnicity, one-quarter of Pearson Chi-Square cells had expected cell frequencies of fewer than 5 participants. Fisher’s exact test could not be run. The Yates Continuity Correction and the Pearson Chi-Square were equal at 0.734. 

To determine differences in pre- to post-test knowledge of HPV-attributable diseases and prophylaxis between multimodal counseling interventions for HPV vaccination among series non-completers, the change in number of participants making *agree* or *strongly agree* responses to the 11-item knowledge variable subscale was analyzed (Table 2, Table 3 and Table 4). Kruskal-Wallis analysis of variance by ranks (*χ*^2^) of the 18.0% to 19.9% increases in the number of participants with knowledge subscale *agree* or *strongly agree* responses in Groups 2 and 3 indicated statistical significance, *p* = 0.038 (Table 3). Follow-up Mann-Whitney *U* testing with Bonferroni correction, showed Group 2 had statistically significant increases in the number of participants with more than half knowledge subscale *agree* or *strongly agree* responses (*p* = 0.04) as shown in Table 5. Groups 2 and 3 achieved statistically significant knowledge improvement of HPV etiologic role in occurrence of EGW, *p* < 0.001. Group 3 achieved statistically significant knowledge improvement pertaining to the purposes of HPV vaccination, *p* = 0.02. Wilcoxon signed-ranks test identified six knowledge items with statistically significant changes in responses using the range of the 5-point Likert scale from pre-test to post-test, with small or medium effect sizes as shown in Table 6 [32]. 

To determine the effect, if any, of covariates on HPV knowledge and HPV vaccination acceptance, Spearman’s rank correlation coefficient (ρ) was calculated. Unadjusted ρ, demonstrated that parents’ age in years, and US generation status were significantly correlated with increased HPV knowledge (*p* = 0.027 and *p* = 0.032 respectively) and HPV vaccination acceptance (*p* = 0.005 and *p* = 0.032 respectively), resulting in more than half *agree* and *strongly agree* responses (Table 7). The number of doses of HPV vaccine received affected increased HPV vaccination acceptance (*p* = 0.011), while the number of male children a parent had was significantly correlated (*p* = 0.028) with increased HPV knowledge resulting in more than half *agree* and *strongly agree* responses. 

To ascertain differences in reported HPV vaccination acceptance rates of HPV vaccination series non-completers across multimodal counseling interventions, the pre-test to post-test change in the number of participants responding *agree* or *strongly agree* to the 14-item acceptance variable subscale items was analyzed. Kruskal-Wallis analysis of variance by ranks (*χ*^2^) of the 12.2% to 12.6% increases in the number of participants with acceptance subscale *agree* or *strongly agree* responses in Groups 1 and 3 respectively (Table 3) did not demonstrate statistical significance. Wilcoxon signed-ranks test indicated eight HPV vaccination acceptance items with statistically significant changes in responses using the 5-point Likert scale from pre-test to post-test, *p* < 0.001 to *p* = 0.023; however, the effect sizes were small or medium as shown in Table 6 [32]. For Group 1, the audiovisual intervention, there were six such items: “A3. Research improves vaccines”, *p* = 0.006, “A5. HPV vaccination would prevent problems for myself/my child(ren)”, *p* = 0.002, “A7. HPV vaccination before teenage is a good idea”, *p* = 0.002, “A9. If the HPV vaccine were available, I/my child(ren) would be vaccinated against HPV”, *p* = 0.002, “A12. Despite cost I will vaccinate myself/my child(ren)”, *p* < 0.001, and “A13. If my doctor recommends I will vaccinate myself/my child(ren)”, *p* = 0.042. For Group 3, the combined audiovisual and PHEH intervention, there were seven such items: “A3. Research improves vaccines”, *p* = 0.001, “A5. HPV vaccination would prevent problems for myself/my child(ren)”, *p* < 0.001, “A7. HPV vaccination before teenage is a good idea”, *p* = 0.001, “A8. Teenagers should be able to get HPV vaccination without parental consent”, *p* = 0.023, “A11. Will only vaccinate myself/my child(ren) against HPV if required”, *p* = 0.002, “A12. Despite cost I will vaccinate myself/my child(ren)”, *p* < 0.001, and “A13. If my doctor recommends I will vaccinate myself/my child(ren)”, *p* = 0.05. 

## 4. Discussion

The objective of this quantitative comparative online survey-based study was to evaluate whether multimodal counseling interventions increase HPV vaccination series non-completers’ knowledge of HPV-attributable disease and acceptance of HPV-attributable disease prophylaxis (vaccination), over a control 14-sentence counseling intervention. Given individuality, it is plausible that different counseling interventions will be necessary to reach different people. Different counseling interventions may have different efficacy. Counseling interventions can increase disease process and vaccination knowledge and acceptance by 9.2 to 15 percentage-points [23,33,34]. However, evidence-based practice and economical resource use require case-by-case counseling intervention outcomes evaluations. Therefore, this head-to-head study design evaluated three counseling interventions with a control standard of care counseling intervention. An effective counseling intervention would simultaneously increase HPV knowledge, and HPV vaccination knowledge and acceptance. Effective counseling intervention use could help raise the U.S.’ female HPV vaccination rate from 32% to an achievable, beneficial, potentially herd immunity sustaining 80%. 

This study found that compared to the control group, all experimental groups showed a greater increase in HPV-attributable disease and HPV vaccination knowledge, *p* = 0.038. The PHEH intervention, and the combined audiovisual and PHEH intervention, raised knowledge of HPV-attributable EGW, *p* < 0.001 (Table 5). The combined audiovisual and PHEH intervention raised knowledge of HPV vaccination purpose, *p* = 0.02 (Table 5). In a primarily Caucasian or Hispanic public health and private pediatric clinic population preintervention parental knowledge that HPV is causative of EGW may range from 16.43 to 36.25% respectively [19].

In this study, preintervention knowledge that EGW is an HPV-attributable disease was 44.82%, and of HPV vaccination purpose 41.57% (Table 4). Thus, this study population’s preintervention knowledge of HPV-attributable EGW (Table 4) is comparable to that of the aforementioned private pediatric clinic population [19]. However, in both instances, preintervention knowledge was noticeably less than that found among community outreach participants in north central Florida, 74.2%, when asked if HPV causes EGW and cervical cancer [35]. Similarly, 74.3% of medical students at a midwestern U.S. medical school were aware that HPV vaccination protects against EGW, and 91.1% were aware that HPV vaccination protects against cervical cancer, both of which represent higher background HPV knowledge than evident in this study’s population [18].

The combined PHEH and audiovisual achieved increased HPV vaccination acceptance for seven items, *p* < 0.001 to *p* = 0.023 (Table 6). The audiovisual intervention achieved increased HPV vaccination acceptance for six items, *p* < 0.001 to *p* = 0.006 (Table 6). Parents were more likely to accept offers of HPV vaccination for a child than were childless young adults for themselves (68.2% and 52.9%). Particularly, parents of more sons than daughters were more likely to accept HPV vaccination. While previous investigators excluded fathers and young male adults from their studies, or had low inclusion of fathers, such as 9% of the sample, in this study fathers and young male adults were 18% and 21% respectively of the sample [24,28,36,37]. The inclusion of 39% male participants may have played into the finding that parents of sons retained the most information about HPV.

The finding that generational status remote from an immigration event is associated with increased knowledge and acceptance of HPV is contrary to Bair et al.’s finding that temporal proximity to an immigration event increased the likelihood to accept vaccination [38]. Yet, these findings are consistent with the literature on older adults and health promotion. Earlier studies found older adults were more likely to participate in health promoting behaviors than were younger adults [39,40]. The association between increased likelihood to accept HPV vaccination and generational status remote from an immigration event is consistent with foreign-born persons living in the U.S. having lower HPV vaccination initiation rates [41].

The findings are both statistically and clinically significant. Clinically, the findings suggest that EGW prevention may motivate HPV vaccination acceptance, and that older parents are more accepting of HPV vaccination than are childless young adults. Parents of male children retained the most knowledge information about HPV vaccination. Given that less intervention is needed to move a person from contemplation to action, than from pre-contemplation to action, participating parents of male children may have been in a contemplative state about HPV, whereas young adults and parents of daughters could have been pre-contemplative [42]. Additionally, as proportionately more parents than childless young adults were initially accepting of HPV vaccination, adults overall may be more likely to be contemplative towards HPV vaccination than childless young adults. For different age groups, different knowledge and acceptance factors, equivalent to pros and cons of vaccination can facilitate a movement to action [42]. Contemplative male participants are consistent with Patel et al., who found 57.9% of males to be contemplative, but inconsistent with Perez et al., who found at least 77.9% of parents of sons to be pre-contemplative of male HPV vaccination [43,44]. Nonetheless, knowledge and awareness increasing educational counseling interventions are targeted for precontemplation and contemplation stages of behavioral change, making parents and young male adults the best targets for the multimodal counseling interventions used in this study [45].

Targeted counseling for HPV vaccination acceptance has demonstrated ability to increase HPV vaccination acceptability [24,26,33,35]. Gain-framed counseling can convince mothers of sons to consider HPV vaccination [46]. Previous investigators had found cervical cancer prevention was a greater motivator for HPV vaccination acceptance than prevention of a sexually transmitted infection [47]. Yet, this study found that EGW, a visible manifestation of an HPV-attributable STI was the most easily communicated HPV-attributed disease. Therefore, clinicians and health promotion initiatives could target households with male children and older parents for HPV vaccination promotion. Clinically, health care providers should realize young adults, households with more female than male children, and persons associated with a proximate American immigration event who have yet to accept HPV vaccination may be pre-contemplative regarding HPV vaccination. In that case, more effort will be needed to make significant gains in HPV and HPV vaccination knowledge and acceptance. Well-designed handouts can be as effective, or more effective than an audiovisual presentation to help to shift stage of change towards contemplation, potentially reducing counseling costs.

There were several limitations to this study. The composite 25-item demographics questionnaire combined with a repeated 49-item survey instrument could have been excessively long [23,24,36,48]. Long surveys may incur diminishing returns with subject fatigue contributing to satisficing behavior and poorer quality responses [49]. Despite the survey’s length potentially important questions such as sexual orientation and unmarried persons’ intimate relationship status, which affects perceived HPV vaccination need were omitted [9,50]. The power to detect differences or adequately describe the target population was reduced by the small sampling of non-Hispanic mixed race, non-Hispanic other, and Hispanic other groups, a smaller proportion of Medicaid participants in the audiovisual intervention Group 1, and of vaccination refusers in the Control group. 

Moreover, this consumer-based online study population is different from random-dial telephone HPV surveys populations, and college-student or other populations completing online surveys. Selection or response bias occurring with choosing which email to respond to may be different from that occurring when choosing to answer a telephone call. As response, not invitation tracking was used, the rates of invitations per respondent and per included respondent are unknown. Different self-selected populations may have different underlying interest in and knowledge of the topic being surveyed [18,35]. Contrary to the literature, neither morality nor religiosity affected HPV vaccination acceptance [26,27,51]. Of course, this finding should be interpreted cautiously due to the sparse representation of several religious categories. Unlike Curtis et al., neither health care provider recommendation, income, residence location, the setting in which vaccines are received, nor race significantly affected HPV vaccination acceptance [52]. Also, contrary to the literature, education did not affect HPV vaccine acceptability [41,45,52,53]. This consumer-based online study is further limited by the lack of provider- or pharmacy-verified vaccination histories. Participant provided HPV-vaccination status could reflect recall and social desirability biases [45].

The audiovisual choice may have not been as well received as the PHEH, reflected in greater response to the latter than the former. A combination of survey length and audiovisual choice may account for the combined audiovisual and PHEH Group 3 not standing out from the PHEH Group 2 as the most effective intervention. Future studies could be conducted with a larger proportion of over 40-years old; non-Hispanic mixed race, non-Hispanic other, and Hispanic other racial or ethnic groups to improve external generalization. A separate study evaluating audiovisual presentations to determine the most effective audiovisual could improve HPV vaccination counseling. Given a potential 23% cost differential in using electronic instead of print counseling materials, increased comparative audiovisual effectiveness is essential for audiovisual counseling use [25].

## 5. Conclusions

The objective of this quantitative comparative online study was to evaluate whether multimodal counseling interventions increase HPV vaccination series non-completers’ knowledge of HPV-attributable disease and acceptance of HPV-attributable disease prophylaxis (vaccination), over a control 14-sentence counseling intervention. The selected consumer-based online survey population has different characteristics than a random-dialed telephone or college-based online survey population. The results showed that some disease and vaccination-specific information could be successfully communicated in the online format yielding changed perceptions. Foremost, that HPV causes EGW, and that HPV vaccination prevents HPV-attributable diseases and their sequelae were better conveyed by the combined audiovisual presentation and PHEH than the control 14-sentence counseling intervention alone.

## Figures and Tables

**Table 1:** Independent variables randomization validation to group assignment.

Independent Variables	Control		Exp. Gp. 1		Exp. Gp. 2		Exp. Gp. 3		*N*	Valid	Chi-	Fischer’s
	*n*	*%*	*n*	*%*	*n*	*%*	*n*	*%*		%	Square	Exact Test
Age (years)											0.211	
Young adults 19–26	138	25.3	125	22.9	134	24.6	148	27.2	545	49.1		
Parents 27 and older	139	24.6	160	28.4	127	22.5	138	24.5	564	50.9		
Biologic sex											0.369	
Female	151	23.9	157	24.8	160	25.3	165	26.1	633	57.1		
Male	126	26.5	128	26.9	101	21.2	121	25.4	476	42.9		
Sex of child(ren)											0.225	
Female and male	45	19.5	65	28.6	59	25.5	61	26.4	231	41.0		
Male	51	30.0	51	30.0	32	18.8	36	21.2	170	30.1		
Female	43	26.4	43	26.4	36	22.1	41	25.2	163	28.9		
Marital Status												<0.001
Married	131	25.5	138	26.9	126	24.6	118	23.0	513	46.3		
Single, never married	110	27.1	87	21.4	100	24.6	109	26.8	406	36.6		
Long-term relationship	18	17.1	28	26.7	23	21.9	36	34.3	105	9.5		
Separated, divorced, widowed	12	18.5	26	40.0	10	15.4	17	26.2	65	5.8		
Common law marriage	6	30.0	6	30.0	2	10.0	6	30.0	20	1.8		
Household size											0.976	
One person	25	28.1	25	28.1	15	16.9	24	27.0	89	8.0		
Two persons	40	22.9	46	26.3	41	23.4	48	27.4	175	15.8		
Three persons	82	24.7	85	25.6	76	22.9	89	26.8	332	29.9		
Four persons	77	26.1	71	24.1	72	24.4	75	25.4	295	26.6		
Five persons	30	23.8	31	24.6	33	26.2	32	25.4	126	11.4		
Six or more persons	23	25.0	27	29.3	24	26.1	18	19.6	92	8.3		
Household children												0.001
None	3	12.5	11	45.8	3	12.5	7	29.2	24	2.2		
One child	178	26.3	164	24.2	152	22.4	184	27.1	678	61.1		
Two children	59	21.1	83	29.6	64	22.9	74	26.4	280	25.2		
Three children	24	29.3	14	17.1	28	34.1	16	19.5	82	7.4		
Four children	9	36.0	3	12.0	10	40.0	3	12.0	25	2.3		
Five or more children	4	20.0	10	50.0	4	20.0	2	10.0	20	1.8		
Race and ethnicity											0.734 *^a^*	cannot
White, non-Hispanic	173	20.0	200	27.5	174	23.9	180	24.8	727	65.6		compute
Hispanic, White	34	27.9	30	24.6	30	24.6	28	23.0	122	11.0		
African-American, non-Hispanic	28	30.0	24	22.2	20	18.5	36	33.3	108	9.7		
Asian, non-Hispanic	19	30.0	19	25.0	15	19.7	23	30.3	76	6.9		
Hispanic, non-White	10	29.4	7	20.6	7	20.6	10	29.4	34	3.1		
Mixed race, non-Hispanic	7	28.0	3	12.0	9	36.0	6	24.0	25	2.3		
Other, non-Hispanic	5	38.5	1	7.7	5	38.5	2	15.4	13	1.2		
Hispanic, Other	1	25.0	1	25.0	1	25.0	1	25.0	4	0.4		
Religion												<0.001
Other Christian	58	22.6	62	24.1	60	23.3	77	30.0	257	23.2		
None	68	28.0	65	26.7	52	21.4	58	23.9	243	21.9		
Catholicism	56	28.3	50	25.3	52	26.3	40	20.2	198	17.9		
Baptist	33	21.4	42	27.3	33	21.4	46	29.9	154	13.9		
Protestantism	24	24.7	25	25.8	27	27.8	21	21.6	97	8.7		
Other	21	22.3	21	22.3	23	24.5	29	30.9	94	8.5		
Mormonism	6	27.3	7	31.8	6	27.3	3	13.6	22	2.0		
Jewish	6	33.3	4	22.2	3	16.7	5	27.8	18	1.6		
Buddhism	3	21.4	5	35.7	2	14.3	4	28.6	14	1.3		
Islam	2	16.7	4	33.3	3	25.0	3	25.0	12	1.1		
Born-again or evangelical Christian											0.282	
Yes	66	22.8	73	25.2	80	27.6	71	24.5	290	26.1		
No	211	25.8	212	25.9	181	22.1	215	26.3	819	73.9		
Frequency of religious services											0.835	
Rarely or never	121	25.3	127	26.6	105	22.0	125	26.2	478	43.1		
A few times annually	63	30.1	49	23.4	47	22.5	50	23.9	209	18.8		
1–3 times a month	31	20.8	39	26.2	38	25.5	41	27.5	149	13.4		
Once weekly	44	22.3	51	25.9	49	24.9	53	26.9	197	17.8		
More than once weekly	18	23.7	19	25.0	22	28.9	17	22.4	76	6.9		
Political leaning											0.241	
Very conservative	42	25.9	51	31.5	35	21.6	34	21.0	162	14.6		
Somewhat conservative	51	24.8	55	26.7	43	20.9	57	27.7	206	18.6		
Middle of the road	93	21.3	111	25.4	112	25.6	121	27.7	437	39.4		
Somewhat liberal	61	30.5	40	20.0	49	24.5	50	25.0	200	18.0		
Very liberal	30	28.8	28	26.9	22	21.2	24	23.1	104	9.4		
Education level											0.448	
Less than high school	2	40.0	1	20.0	1	20.0	1	20.0	5	0.5		
Some high school	7	20.6	9	26.5	9	26.5	9	26.5	34	3.1		
9th grade	2	15.4	4	30.8	3	23.1	4	30.8	13	1.2		
10th grade	2	22.2	3	33.3	2	22.2	2	22.2	9	0.8		
11th grade	3	25.0	2	16.7	4	33.3	3	25.0	12	1.1		
Completed high school/GED	59	25.4	60	25.9	61	26.3	52	22.4	232	20.9		
Some college	78	23.0	89	26.3	73	21.5	99	29.2	339	30.6		
College graduate	95	25.3	88	23.4	100	26.6	93	24.7	376	33.9		
Graduate school	36	29.3	38	30.9	17	13.8	32	26.0	123	11.1		
Household income											0.275	
Less than $20,000	50	26.2	46	24.1	49	25.7	46	24.1	191	17.2		
$20,000–$39,999	52	26.1	43	21.6	52	26.1	52	26.1	199	17.9		
$40,000–$49,999	38	25.2	41	27.2	32	21.2	40	26.5	151	13.6		
$50,000–$59,999	49	29.0	36	21.3	42	24.9	42	24.9	169	15.2		
$60,000–$100,000	48	20.3	76	32.2	49	20.8	63	26.7	236	21.3		
More than $100,000	28	28.9	30	30.9	16	16.5	23	23.7	97	8.7		
Declined to answer	12	18.2	13	19.7	21	31.8	20	30.3	66	6.0		
Employment status							0.441				0.441	
Full time employee	111	24.4	115	25.3	113	24.8		25.5	455	41.0		
Homemaker	54	25.0	60	27.8	56	25.9		21.3	216	19.5		
Part time employee	52	28.1	38	20.5	43	23.2		28.1	185	16.7		
Full time student, not working	26	19.8	38	29.0	26	19.8		31.3	131	11.8		
Unemployed	34	27.9	34	27.9	23	18.9		25.4	122	11.0		
Location of home							0.923				0.923	
Urban	85	24.5	90	26.0	81	23.4		26.0	346	31.2		
Suburban	126	24.3	134	25.8	119	22.9		27.0	519	46.8		
Rural	66	27.0	61	25.0	61	25.0		23.0	244	22.0		
Type of health insurance							0.038 *				0.038 *	
Private	146	22.6	180	27.8	143	22.1		27.5	647	58.3		
Medicaid	80	29.4	53	19.5	74	27.2		23.9	272	24.5		
Other	51	26.8	52	27.4	44	23.2		22.6	190	17.1		
Vaccination setting							0.863				0.863	
Healthcare provider’s office	195	25.0	204	26.2	183	23.5		25.3	779	70.2		
Hospital	42	25.8	38	23.3	43	26.4		24.5	163	14.7		
Community clinic	23	21.1	31	28.4	22	20.2		30.3	109	9.8		
County clinic	17	29.3	12	20.7	13	22.4		27.6	58	5.2		
Frequency of healthcare visits							0.579				0.579	
Annual checkup	130	23.0	15	27.0	134	23.8		26.2	564	50.9		
Only when sick	119	26.1	115	25.2	104	22.8		25.9	456	41.1		
Other	28	31.5	18	20.2	23	25.8		22.5	89	8.0		
Regular healthcare provider							0.154				0.154	
Yes	210	23.6	232	26.1	209	23.5		26.8	889	80.2		
No	67	30.5	53	24.1	52	23.6		21.8	220	19.8		
US Generation *^b^* (*n* = 1108)							0.321				0.321	
First generation	38	30.9	30	24.4	23	18.7		26.0	123	12.1		
Second generation	38	28.1	26	19.3	31	23.0		29.6	135	13.3		
Third generation	180	23.7	204	26.8	178	23.4		26.1	760	74.7		
Know someone with a STD							0.625				0.625	
Yes	101	24.8	97	23.8	103	25.3		26.0	407	36.7		
No	176	25.1	188	26.8	158	22.5		25.6	702	63.3		
Personally had a STD							0.555				0.555	
Yes	27	23.3	25	21.6	32	27.6		27.6	116	10.5		
No	250	25.2	260	26.2	229	23.1		25.6	993	89.5		
Ever heard of HPV							0.850				0.850	
Yes	221	25.0	230	26.0	210	23.8		25.2	884	79.7		
No	56	24.9	55	24.4	51	22.7		28	225	20.3		
Ever heard of HPV vaccine							0.684				0.684	
Yes	214	25.3	215	25.4	204	24.1		25.1	845	76.2		
No	63	23.9	70	26.5	57	21.6		28.0	264	23.8		
Offered HPV vaccine for child(ren) or self							0.566				0.566	
Yes	82	22.7	92	25.5	92	25.5		26.3	361	32.6		
No	195	26.1	193	25.8	169	22.6		25.5	748	67.4		
Accepted HPV vaccine *^c^* (*n* = 218)											0.029 *	
Yes	61	27.1	54	24.0	47	20.9	63	28.0	218	60.4		
No	26	17.3	38	25.3	49	32.7	37	24.7	143	39.6		
HPV vaccine doses *^d^* (*n* = 219)									219		0.054	
None	3	25.0	1	8.3	6	50.0	2	16.7	12	5.5		
One dose	39	26.9	40	27.6	22	15.2	44	30.3	145	66.21		
Two doses	16	25.8	54	21.0	18	29.0	15	24.2	62	28.3		

*^a^* 8 cells (25%) have expected count less than 5, Yates continuity correction. 734 = Pearson Chi-Square. *^b^* (*n* = 1108) as there were 91 foreign born participants. *^c^* (*n* = 218) is the number of included participants who were offered and accepted HPV vaccine, but who did not complete the 3-dose HPV vaccination series. *^d^* (*n* = 219) includes one subject who was not offered, but received HPV vaccine. * denotes statistical significance the alpha = *p* = 0.05 level. Due to rounding percentages may not add up to 100. Exp. Gp. = Experimental Group; GED = General education development certificate; HPV = Human papilloma virus; STD = sexually transmitted disease; US = United States of America.

**Table 2:** Intervention group scale summary: More than half post-test responses showing HPV Knowledge and HPV vaccination acceptance.

Scale	Control *^a^* (*n* = 277)				Exp. Group 1 *^b^* (*n* = 285)				Exp. Group 2 *^c^* (*n* = 261)				Exp. Group 3 *^d^* (*n* = 286)				All Groups *^e^* (*n* = 1109)			
	Pre	Post	Diff.	%	Pre	Post	Diff.	%	Pre	Post	Diff.	%	Pre	Post	Diff.	%	Pre	Post	Diff.	%
Knowledge subscale	147	171	24	8.7	154	194	40	14.0	136	183	47	18.0	144	201	57	19.9	581	749	168	15.1
Acceptance subscale	87	113	26	9.4	105	141	36	12.6	95	116	21	8.0	103	138	35	12.2	390	508	118	10.6

*^a^* (*n* = 277) is the number of participants in the Control group. *^b^* (*n* = 285) is the number of participants in Experimental Group 1. *^c^* (*n* = 261) is the number of participants in Experimental Group 2. *^d^* (*n* = 286) is the number of participants in Experimental Group 3. *^e^* (*n* = 1109) is the number of participants who completed the survey through the quantitative post-test; the sum of the participants in each of the intervention groups. Diff = pre- to post-test change; Exp. = Experimental; Pre = Pre-test; Post = Post-test.

**Table 3:** Intervention group scale analysis: More than half post-test responses showing HPV Knowledge and HPV vaccination acceptance.

Scale	Control *^a^* (*n* = 277)	Exp. Group 1 *^b^* (*n* = 285)	Exp. Group 2 *^c^* (*n* = 261)	Exp. Group 3 *^d^* (*n* = 286)	Across/between Groups Comparison		
	Kruskal-Wallis Mean Rank	Kruskal-Wallis Mean Rank	Kruskal-Wallis Mean Rank	Kruskal-Wallis Mean Rank	Chi-Square	*df*	*p* Value
Knowledge Subscale							
Change > half overall Responses *Agree* or *Strongly Agree*	517.89	540.04	585.31	578.18	8.443	3	0.038 *
3. HPV causes genital warts	514.47	506.13	604.96	597.37	34.887	3	<0.001 **
9. I know what the HPV vaccine is for	522.18	548.34	561.97	587.06	8.556	3	0.036 *
Acceptance Subscale							
Change > half Overall Responses *Agree* or *Strongly Agree*	531.84	571.39	550.14	565.53	2.628	3	0.453

*^a^* (*n* = 277) is the number of participants in the Control group. *^b^* (*n* = 285) is the number of participants in Experimental Group 1. *^c^* (*n* = 261) is the number of participants in Experimental Group 2. *^d^* (*n* = 286) is the number of participants in Experimental Group 3. * denotes significant 2-tailed correlation at the alpha = *p* = 0.05 level. *df =* degrees of freedom; ** denotes significant 2-tailed correlation at the alpha = *p* = 0.01 level; ; Exp. = Experimental.

**Table 4:** Intervention group knowledge item summary: Per group respondents with more than half post-test responses showing HPV Knowledge.

Knowledge Subscale Items	Control *^a^* (*n* = 277)				Exp. Group 1 *^b^* (*n* = 285)				Exp. Group 2 *^c^* (*n* = 261)				Exp. Group 3 *^d^* (*n* = 286)				Overall *^e^* (*n* = 1109)			
	Pre-test		Post-test		Pre-test		Post-test		Pre-test		Post-test		Pre-test		Post-test		Pre-test		Post-test	
	No.	%	No.	%	No.	%	No.	%	No.	%	No.	%	No.	%	No.	%	No.	%	No.	%
3. HPV causes genital warts	119	42.96	127	45.85	137	48.07	140	49.13	117	44.83	173	66.28	124	43.36	180	62.94	497	44.82	620	55.91
9. I know what the HPV vaccine is for	103	37.18	149	53.79	135	47.37	197	69.12	106	40.61	170	65.13	117	40.91	201	70.28	461	41.57	717	64.65

*^a^* (*n* = 277) is the number of participants in the Control group. *^b^* (*n* = 285) is the number of participants in Experimental Group 1. *^c^* (*n* = 261) is the number of participants in Experimental Group 2. *^d^* (*n* = 286) is the number of participants in Experimental Group 3. *^e^* (*n* = 1109) is the number of participants who completed the survey through the quantitative post-test; the sum of the participants in each of the intervention groups. Exp. = Experimental; * denotes significant 2-tailed correlation at the alpha = *p* = 0.05 level. ** denotes significant 2-tailed correlation at the alpha = *p* = 0.01 level.

**Table 5:** Intervention group summary: Changes in responses on the HPV knowledge and HPV vaccination acceptance subscales.

Subscale Item	Paired Groups for Comparison Group: Intervention	*n*	Mean Rank	Mann-Whitney *U*	*Z*	*p* Value	Bonferroni Corrected *p* Value
Change in *Agree* and *Strongly Agree*	Control group: Control	277	276.37				
Knowledge Responses over 50% mark	Exp. Group 1: Control and Audiovisual	285	286.48	38,052.00	−0.750	0.453	
	Control group: Control	277	253.06				
	Exp. Group 2: Control and Handout	261	286.94	31,595.50	−2.573	0.010	0.04 *
	Control group: Control	277	266.46				
	Exp. Group 3: Control, Audiovisual and Handout	286	297.05	35,305.50	−2.272	0.023	0.092
K3. HPV causes genital warts	Control group: Control	277	283.78				
	Exp. Group 1: Control and Audiovisual	285	279.28	38,840.50	−0.437	0.662	
	Control group: Control	277	247.98				
	Exp. Group 2: Control and Handout	261	292.34	30,188.50	−4.193	<0.001	<0.001 **
	Control group: Control	277	260.70				
	Exp. Group 3: Control, Audiovisual and Handout	286	302.63	33,711.00	−3.778	<0.001	<0.001 **
K9. I know what the HPV vaccine is for	Control group: Control	277	274.73				
	Exp. Group 1: Control and Audiovisual	285	288.08	37,597.50	−1.181	0.238	
	Control group: Control	277	260.10				
	Exp. Group 2: Control and Handout	261	279.47	33,546.00	−1.741	0.082	
	Control group: Control	277	265.35				
	Exp. Group 3: Control, Audiovisual and Handout	286	298.13	34,998.50	−2.832	0.005	0.02 *
Change in *Agree* and *Strongly Agree*	Control group: Control	277	271.62				
Acceptance Responses over 50% mark	Exp. Group 1: Control and Audiovisual	285	291.11	36,734.50	−1.439	0.150	
	Control group: Control	277	264.85				
	Exp. Group 2: Control and Handout	261	274.43	34,861.00	−0.725	0.468	
	Control group: Control	277	273.38				
	Exp. Group 3: Control, Audiovisual and Handout	286	290.35	37,222.00	−1.253	0.210	

Exp. = Experimental; K = Knowledge subscale item; * denotes significant 2-tailed correlation at the alpha = *p* = 0.05 level using the mathematically equivalent Bonferroni adjustment of calculated *p* value × 4. ** denotes significant 2-tailed correlation at the alpha = *p* = 0.01 level using the mathematically equivalent Bonferroni adjustment of calculated *p* value × 4.

**Table 6:** Per question summary: Wilcoxon signed ranks test analysis of pre-test to post-test response changes.

**Question**	**Control Group *^a^* (*n* = 277), *^A^* (*N* = 554)**					**Experimental Group 1 *^b^* (*n* = 285), *^B^* (*N* = 570)**				
	**Median Pre-test**	**Median Post-test**	***Z***	***p***	**Effect Size *r***	**Median Pre-test**	**Median Post-test**	***Z***	***p***	**Effect Size *r***
K1. HPV is a STD	Agree (4)	Agree (4)	−3.944	<0.001 **	0.168 ^s^	Agree (4)	Agree (4)	−3.239	0.001 **	0.136 ^s^
K2. Condoms prevent HPV	Agree (4)	Agree (4)	−1.563	0.118	0.066	Agree (4)	Neutral (3)	−0.134	0.893	0.006
K3. HPV causes genital warts	Neutral (3)	Neutral (3)	−0.990	0.322	0.042	Neutral (3)	Neutral (3)	−0.102	0.919	0.004
K4. People with HPV may be asymptomatic	Agree (4)	Agree (4)	−1.823	0.068	0.077	Agree (4)	Agree (4)	−0.876	0.381	0.037
K5. HPV causes sterility	Neutral (3)	Neutral (3)	−3.030	0.002 **	0.129 ^s^	Neutral (3)	Neutral (3)	−1.471	0.141	0.062
A1. Worry that I/my child(ren)can get HPV	Neutral (3)	Neutral (3)	−3.879	<0.001 **	0.165 ^s^	Neutral (3)	Neutral (3)	−4.078	<0.001 **	0.171 ^s^
K6. HPV causes cervical cancer	Agree (4)	Agree (4)	−3.724	<0.001 **	0.158 ^s^	Agree (4)	Agree (4)	−4.336	<0.001 **	0.182 ^s^
K7. Treatment of HPV is painful	Neutral (3)	Neutral (3)	−1.576	0.115	0.067	Neutral (3)	Neutral (3)	−0.858	0.391	0.036
K8. Required vaccines protect from getting disease from unvaccinated persons	Agree (4)	Agree (4)	−1.324	0.186	0.056	Agree (4)	Agree (4)	−3.292	0.001 **	0.138 ^s^
K9. I know what the HPV vaccine is for	Neutral (3)	Agree (4)	−6.689	<0.001 *	0.284 ^m^	Neutral (3)	Agree (4)	−6.924	<0.001 **	0.029 ^s^
K10. Genital warts make it hard to have a sexual partner	Agree (4)	Agree (4)	−0.845	0.398	0.036	Agree (4)	Agree (4)	−1.289	0.197	0.054
K11. Children should only be vaccinated for serious diseases	Neutral (3)	Neutral (3)	−0.524	0.600	0.022	Neutral (3)	Neutral (3)	−0.320	0.749	0.013
A2. Vaccines that have been used awhile are more trustworthy	Agree (2)	Agree (2)	−1.547	0.122	0.066	Agree (2)	Agree (2)	−1.068	0.285	0.045
A3. Research improves vaccines	Agree (4)	Agree (4)	−1.122	0.262	0.048	Agree (4)	Agree (4)	−2.763	0.006 **	0.116 ^s^
A4. Healthy children do not need vaccines	Disagree (4)	Disagree (4)	−1.506	0.132	0.064	Disagree (4)	Disagree (4)	−1.898	0.058	0.079
A5. HPV vaccination would prevent problems for myself/my child(ren)	Neutral (3)	Agree (4)	−3.088	0.002 **	0.131 ^s^	Agree (4)	Agree (4)	−3.130	0.002 **	0.131 ^s^
A6. Giving a new vaccine is like performing an experiment	Neutral (3)	Neutral (3)	−0.176	0.860	0.007	Neutral (3)	Neutral (3)	−0.071	0.943	0.003
A7. HPV vaccination before teenage is a good idea	Neutral (3)	Neutral (3)	−4.118	<0.001 **	0.175 ^s^	Neutral (3)	Neutral (3)	−3.162	0.002 **	0.132 ^s^
A8. Teenagers should be able to get HPV vaccination without parental consent	Neutral (3)	Neutral (3)	−1.243	0.214	0.053	Neutral (3)	Neutral (3)	−0.958	0.338	0.040
A9. If the HPV vaccine were available, I/my child(ren) would be vaccinated against HPV	Neutral (3)	Neutral (3)	−1.250	0.211	0.053	Neutral (3)	Neutral (3)	−2.748	0.006 **	0.115 ^s^
A10. Vaccines are painful, so I would not vaccinate myself/my child(ren)	Neutral (3)	Disagree (4)	−0.707	0.480	0.030	Disagree (4)	Disagree (4)	−1.110	0.267	0.046
A11. Will only vaccinate myself/my child(ren) against HPV if required	Neutral (3)	Neutral (3)	−1.968	0.049 *	0.084 ^s^	Neutral (3)	Neutral (3)	−1.174	0.240	0.049
A12. Despite cost I will vaccinate myself/my child(ren)	Neutral (3)	Neutral (3)	−2.332	0.020 *	0.099 ^s^	Neutral (3)	Neutral (3)	−3.623	<0.001 **	0.152 ^s^
A13. If my doctor recommends I will vaccinate myself/my child(ren)	Agree (4)	Agree (4)	−0.555	0.579	0.024	Agree (4)	Agree (4)	−2.035	0.042 *	0.085 ^s^
A14. When I decide to vaccinate myself/my child(ren) it will be done	Agree (4)	Agree (4)	−0.091	0.927	0.004	Agree (4)	Agree (4)	−1.236	0.217	0.052
**Question**	**Control Group *^c^* (*n* = 261), *^C^* (*N* = 522)**					**Experimental Group 1 *^d^* (*n* = 286), *^D^* (*N* = 572)**				
	**Median Pre-test**	**Median Post-test**	***Z***	***p***	**Effect Size *r***	**Median Pre-test**	**Median Post-test**	***Z***	***p***	**Effect Size *r***
K1. HPV is a STD	Agree (4)	Agree (4)	−5.952	<0.001 **	0.260^m^	Agree (4)	Agree (4)	−5.569	<0.001 **	0.233 ^m^
K2. Condoms prevent HPV	Agree (4)	Agree (4)	−1.498	0.134	0.066	Neutral (3)	Neutral (3)	−0.007	0.995	0.000 ^e^
K3. HPV causes genital warts	Neutral (3)	Agree (4)	−5.932	<0.001 **	0.260 ^m^	Neutral (3)	Agree (4)	−4.880	<0.001 **	0.204 ^m^
K4. People with HPV may be asymptomatic	Agree (4)	Agree (4)	−2.329	0.020 *	0.102 ^s^	Agree (4)	Agree (4)	−4.131	<0.001 **	0.173 ^s^
K5. HPV causes sterility	Neutral (3)	Neutral (3)	−0.754	0.451	0.033	Neutral (3)	Neutral (3)	−1.619	0.105	0.068
A1. Worry that I/my child(ren) can get HPV	Neutral (3)	Neutral (3)	−3.471	0.001 **	0.152 ^s^	Neutral (3)	Neutral (3)	−5.296	<0.001 **	0.221 ^m^
K6. HPV causes cervical cancer	Agree (4)	Agree (4)	−4.662	<0.001 **	0.204 ^m^	Agree (4)	Agree (4)	−7.387	<0.001 **	0.309 ^m^
K7. Treatment of HPV is painful	Neutral (3)	Neutral (3)	−1.245	0.213	0.054	Neutral (3)	Neutral (3)	−1.326	0.185	0.055
K8. Required vaccines protect from getting disease from unvaccinated persons	Agree (4)	Agree (4)	−2.836	0.005 **	0.124 ^s^	Agree (4)	Agree (4)	−2.166	0.030 *	0.091
K9. I know what the HPV vaccine is for	Neutral (3)	Agree (4)	−7.145	<0.001 **	0.313 ^m^	Neutral (3)	Agree (4)	−8.532	<0.001 **	0.357 ^m^
K10. Genital warts make it hard to have a sexual partner	Agree (4)	Agree (4)	−2.784	0.005 **	0.122 ^s^	Agree (4)	Agree (4)	−1.724	0.085	0.072
K11. Children should only be vaccinated for serious diseases	Neutral (3)	Neutral (3)	−0.182	0.855	0.008	Neutral (3)	Neutral (3)	−1.473	0.141	0.062
A2. Vaccines that have been used awhile are more trustworthy	Agree (2)	Agree (2)	−0.708	0.479	0.031	Disagree (4)	Disagree (4)	−1.266	0.206	0.053
A3. Research improves vaccines	Agree (4)	Agree (4)	−1.006	0.315	0.044	Agree (4)	Agree (4)	−3.447	0.001 **	0.144 ^s^
A4. Healthy children do not need vaccines	Disagree (4)	Disagree (4)	−1.321	0.187	0.058	Disagree (4)	Disagree (4)	−0.638	0.523	0.027
A5. HPV vaccination would prevent problems for myself/my child(ren)	Agree (4)	Agree (4)	−3.530	<0.001 **	0.154 ^s^	Neutral (3)	Agree (4)	−5.684	<0.001 **	0.238 ^m^
A6. Giving a new vaccine is like performing an experiment	Neutral (3)	Neutral (3)	−0.645	0.519	0.028	Neutral (3)	Neutral (3)	−0.858	0.391	0.036
A7. HPV vaccination before Teenage is a good idea	Neutral (3)	Neutral (3)	−3.491	<0.001 **	0.153 ^s^	Neutral (3)	Neutral (3)	−3.182	0.001 **	0.133 ^s^
A8. Teenagers should be able to Get HPV vaccination without parental consent	Neutral (3)	Neutral (3)	−1.867	0.062	0.082	Neutral (3)	Neutral (3)	−2.277	0.023 *	0.095 ^s^
A9. If the HPV vaccine were available, I/my child(ren) would be vaccinated against HPV	Neutral (3)	Neutral (3)	−0.593	0.553	0.026	Neutral (3)	Neutral (3)	−1.929	0.054	0.081
A10. Vaccines are painful, so I Would not vaccinate myself/my child(ren)	Disagree (4)	Disagree (4)	−1.653	0.098	0.072	Agree (2)	Disagree (4)	−0.376	0.707	0.016
A11. Will only vaccinate myself/my child(ren) against HPV if required	Neutral (3)	Neutral (3)	−0.185	0.854	0.008	Neutral (3)	Neutral (3)	−3.096	0.002 **	0.130 ^s^
A12. Despite cost I will vaccinate myself/my child(ren)	Neutral (3)	Neutral (3)	−2.761	0.006 **	0.121 ^s^	Neutral (3)	Agree (4)	−4.747	<0.001 **	0.200 ^s^
A13. If my doctor recommends I will vaccinate myself/my child(ren)	Agree (4)	Agree (4)	−2.237	0.025 *	0.098 ^s^	Agree (4)	Agree (4)	−2.833	0.005 **	0.118 ^s^
A14. When I decide to vaccinate myself/my child(ren) it will be done	Agree (4)	Agree (4)	−2.928	0.003 **	0.128 ^s^	Agree (4)	Agree (4)	−0.698	0.485	0.030

*^a^* (*n* = 277) is the number of participants in the Control group. *^A^* (*N* = 554) is twice the number of participants in the Control group, accounting for the number of pre- and post-tests. *^b^* (*n* = 285) is the number of participants in Experimental Group 1. *^B^* (*N* = 554) is twice the number of participants in Experimental Group 1, accounting for the number of pre- and post-tests. *^c^* (*n* = 261) is the number of participants in Experimental Group 2. *^C^* (*N* = 554) is twice the number of participants in Experimental Group 2, accounting for the number of pre- and post-tests. *^d^* (*n* = 286) is the number of participants in Experimental Group 3. *^D^* (*N* = 554) is twice the number of participants in Experimental Group 3, accounting for the number of pre- and post-tests. A = Acceptance subscale item; K = Knowledge subscale item; e = actual r value is .0003; m = medium effect size; s = small effect size. * denotes significant 2-tailed correlation at the alpha = *p* = 0.05 level. ** denotes significant 2-tailed correlation at the alpha = *p* = 0.01 level.

**Table 7:** Covariates affect HPV knowledge and HPV vaccination acceptance pre-test to post-test change.

Covariate	Knowledge Subscale Change		Acceptance Subscale Change	
	Spearman’s ρ	Significance, 2-Tailed	Spearman’s ρ	Significance, 2-Tailed
Young Adults’ Actual Age in Years	−0.011	0.796	0.073	0.088
Parents’ Actual Age in Years	0.093	0.027 *	0.119	0.005 **
Number of Female Children	0.017	0.690	0.008	0.846
Number of Male Children	0.093	0.028 *	0.017	0.693
Number of HPV Vaccine Doses Child(ren) or Young Adult Received	0.092	0.176	−0.171	0.011 *
Household Size	0.008	0.799	0.041	0.171
Number of Children in the Household	0.016	0.602	0.52	0.084
Generations Born in the US	0.067	0.032 *	0.067	0.032 *
Non-US Born: Years Lived in the US	−0.085	0.425	0.102	0.337
Religious Services Frequency	0.025	0.403	0.026	0.385
Education Level	−0.013	0.661	0.023	0.443
High School non-completer’s Grade	0.042	0.815	0.156	0.379
Income Level	−0.024	0.424	0.021	0.491

Unadjusted data. * denotes significant 2-tailed correlation at the alpha = *p* = 0.05 level. ** denotes significant 2-tailed correlation at the alpha = *p* = 0.01 level.

## Supplementary Materials

The following are available online at www.mdpi.com/2227-9032/5/4/86/s1. Questionnaire S1: Demographic Questionnaire, Questionnaire S2: Young adult HPV Knowledge and Acceptance survey, Questionnaire S3: Parent HPV Knowledge and Acceptance survey, Intervention S1: 14-sentence informational brief, Intervention S2: Public health education handout, Permission S1: A. T. Still University Institutional Review Board, Permission S2: Consent to use the 14-sentence informational brief, Permission S3: Permission to use the audiovisual intervention, Permission S4: Permission to use the public health education handout, Figure S1: Participant demographics: Age by group, Figure S2: Participant demographics: Biologic sex by group, Figure S3: Participant demographics: Children’s biologic sex by group, Figure S4: Participant demographics: Race/ethnicity by group.
