# Supplementary material for: Multimodal Counseling Interventions: Effect on Human Papilloma Virus Vaccination Acceptance
Source: Healthcare (Basel). 2017 Nov 6;5(4):86. doi: 10.3390/healthcare5040086 (PMC5746720; doi:10.3390/healthcare5040086)
Supplement: Supplementary file 1 [file healthcare-05-00086-s001.pdf]

Questionnaire S1: Demographic / Screening Questionnaire

1. What is your age?
   a. For young adults: 19, 20, 21, 22, 23, 24, 25, 26.

      If a young adult, aged 27 or older, or 18 or younger, screened out, exit survey.
   b. For parents: Please write in your exact age.

      If a parent, aged 26 or younger, screened out, exit survey
2. Have you/your child(ren) received three doses of human papilloma virus (HPV) vaccination?

   Yes or no. If yes, screened out, exit survey.
3. What is your biological sex?
   a. Male or female.

      If a parent: Please give the biological sex of your child(ren): Male or female.

      If a parent: Please write in the number of female children.

      Please write in the number of male children.
4. What is your race and ethnicity?

   African American or Black-American. Asian, non-Hispanic. Hispanic, White. Hispanic, non-White. Mixed-race, non-Hispanic. Other, non-Hispanic. Other, Hispanic. White, non-Hispanic.
5. What language do you speak?

   English, Spanish, or other.
   a. If you do not speak English are you able to complete this survey in English, including filling in your opinions in English?

      Yes or no.

If no screened out, exit survey (all free text responses need to be in English).

6. What is your religion?

   Baptist. Buddhism. Catholicism. Jewish. Mormonism. Islam. Protestantism (non-Baptist). Other Christian. None. Other.

7. Are you a born-again or evangelical Christian?

   Yes or no.

8. How often do you attend religious services?

   Rarely or never. Few times a year. 1-3 times a month. Once a week.

   More than once a week.

9. What is your education level?

   No high school. Did some high school (specify grade).

   Completed high school or got my GED. Did some college. College graduate.

   Graduate or professional school.

10. What is your income level?

    $15,000 or less. $15,000 to $30,000. $30,000 to $45,000. $45,000 to $60,000.

    $60,000 to $100,000. More than $100,000. Decline to answer.

11. Is the area in which you live: Urban? Suburban? Rural?

12. What is your employment status?

    Homemaker. Full time employee. Part time employee. Unemployed.

    Full time student, not working.

    Part-time student, not working. Part-time student, working.

13. What is your marital status?

Single, never married. Single, separated. Divorced. Widowed. Married. Common law marriage. Long-term relationship.

14. How many people live in your household?

    One. Two. Three. Four. Five. Six or more.

15. How many children under 18 years old are there in your household?

    None. One. Two. Three. Four. Five or more.

16. What type of health insurance do you have?

    Private. Medicaid. None.

17. Do you have a regular health care provider?

    Yes or no.

18. How often do you see your health care provider?

    Only when sick. Annual checkup. Other.

19. Where do you normally get immunizations?

    Healthcare providers' office. Community clinic. County clinic. Hospital.

20. Do you know someone who has had a sexually transmitted infection (STD)?

    Yes or no.

21. Have you had a sexually transmitted infection (STD)?

    Yes or no.

22. Where were you born?

    a. Born in the U.S.

        a. How many generations of your family have been born in the U.S.?

            First generation. Second generation. Third or higher generation.

    b. Born outside the U.S. (foreign born).

Mexico. Other Central/South America. Asia. Other.

i. If born outside the U.S. (foreign born), how many years have you lived in the U.S.?

Please write in the number of years you have lived in the U.S.

23. What is your political leaning?

    Very conservative. Somewhat conservative. Middle of the road.

    Somewhat liberal. Very liberal.

24. Have you ever heard of the human papilloma virus (HPV)?

    Yes or no.

25. Have you ever heard of the human papilloma virus (HPV) vaccine?

    (This may also be called the Cervarix vaccine or the Gardasil vaccine)

    Yes or no.

26. Have you or your child(ren) been offered human papilloma virus (HPV) vaccine for yourself/your child(ren) by a healthcare provider?

    Yes or no.

    a. If yes, was the vaccine accepted or refused?

       Accepted.

       i. If accepted, how many doses of human papilloma virus (HPV) vaccine did you/your child receive?

          a. None. One. Two. Three.

          b. If three (3) doses of human papilloma virus (HPV) vaccine were received, screened out

       Refused.

Questionnaire S2: Human Papilloma Virus Knowledge and Acceptance Questions for Young Adults

For each of the following statements indicate your opinion on the scale ranging from 1 *(strongly disagree),* 2 *(disagree)*, 3 *(neutral),* 4 *(agree),* and 5 *(strongly agree)* (adapted from Thomas, T. L., Strickland, O., Diclemente, R., & Higgins, M. [2013b]. *Parental human papillomavirus survey* [Database record]. Retrieved from PsycTESTS. http://dx.doi.org/10.1037/t24518-000)

1. HPV is a sexually transmitted disease.
2. Using condoms can prevent HPV.
3. Genital warts are caused by HPV.
4. People with HPV might not have symptoms.
5. HPV makes you unable to have children.
6. I worry that I might get HPV.
7. HPV can cause cervical cancer.
8. Treatment for HPV is painful.
9. Required vaccinations protect me from getting diseases from unvaccinated people.
10. I understand exactly for what the HPV vaccine is used.
11. Having genital warts makes it very difficult to find a sexual partner.
12. People should only get vaccinated for serious diseases.
13. I am more likely to trust vaccinations that have been around awhile.
14. Vaccinations are getting better all the time because of research.
15. Healthy children do not need vaccinations.
16. A vaccine against HPV could prevent future problems for me.
17. Giving me a new vaccine is like performing an experiment on me.

18. Most people I know think vaccinating children with the HPV vaccine before they are teenagers is a good idea.
19. A teenager should be able to get the HPV vaccination without a parent's consent.
20. If this new HPV vaccine was available when I was an infant, I would be vaccinated against HPV infection.
21. Shots are very painful for me so I would rather not get vaccinated.
22. If the new HPV vaccine is not required, I will not get vaccinated.
23. I think that even if the vaccine is expensive I will be able to get vaccinated.
24. Generally I do what my doctor recommends so I will get vaccinated.
25. When I decide to get myself vaccinated, I believe I will be able to get myself vaccinated; in other words, I feel confident I can get myself vaccinated.

For the next two questions indicate your opinion on the scale ranging from 1 *(very unlikely)*, 2 *(somewhat unlikely)*, 3 *(somewhat likely)*, and 4 *(very likely)* (adapted for young adults from Constantine, N. A. & Jerman, P. [2007b]. *HPV vaccination-related interview* [Database record]. Retrieved from PsycTESTS. http://dx.doi.org/10.1037/t09635-000

26. If an HPV vaccine were available, how likely would you have been vaccinated before your 13th birthday? (If the respondent answers question #26 with *very likely*, then question #27 is skipped, and the answer is assumed to be also *very likely)*.
27. If an HPV vaccine were available, how likely would you have been vaccinated before your 16th birthday?
28. What are your reasons for feeling this way about vaccinating yourself? (English free text response limited to 50 words, qualitative question #1)

For each of the following questions select yes or no based on your opinion (adapted for young adults from Morales-Campos, D. Y., Markham, C. M., Peskin, M. F., & Fernandez, M. E. [2013]. Hispanic mothers' and high school girls' perceptions of cervical cancer, human papilloma virus, and the human papilloma virus vaccine [Appendix 1. Focus group guide questions]. *Journal of Adolescent Health, 52*(5), S69-75. http://dx.doi.org/10.1016/j.adohealth.2012.09.020

29. Do you think teen girls could get cervical cancer?
30. Have you heard of the human papillomavirus or HPV?
31. Do you think teenagers could get HPV?
32. Have you heard about the HPV vaccine?

For each of the following questions, please write in your opinion in response to the question asked. (English free text responses limited to 50 words for each response, qualitative questions 2 – 18).

33. What do you think of when you hear the word *cancer*?
34. What do you think of when you hear *cervical cancer*?
35. What do you think causes cervical cancer?
36. Who do you think gets cervical cancer?
37. What are the reasons you believe teen girls could (not) get cervical cancer?
38. If you have heard of the human papillomavirus or HPV, where did you hear about HPV?
39. If you have heard of the human papillomavirus or HPV, what have you heard about HPV?
40. Who do you think gets HPV?

41. What are the reasons you believe teenagers could (or could not) get HPV?
42. If you have heard about the HPV vaccine, what have you heard?
43. If you have heard about the HPV vaccine, where did you hear it?
44. What are your feelings about getting the HPV vaccine?
45. What are the reasons you have these feelings about getting the HPV vaccine?
46. What do you think the good things (benefits) would be of getting the HPV vaccine?
47. What are your worries about getting the HPV vaccine?
48. What might keep you from getting vaccinated?
49. How do you think the cost would affect your decision to get vaccinated?

Survey item analysis categorization

Knowledge items: 1, 2, 3, 4, 5, 7, 8, 9, 10, 11, [12].

Acceptance items: 6, [13], 14, [15], 16, [17], 18, 19, 20, [21], [22], 23, 24, 25.

Post-ARP knowledge items: 29, 30, 31, 32, 33,34,35,36, [37], 38, 39, 40, 41, 42,43.

Post-ARP acceptance items: 26, 27, 28, 44, 45, 46, 47, 48, 49.

For question numbers in brackets a positive response is a negative association for the category. That is a positive response indicates inadequate knowledge or non-acceptance of vaccination.

Questionnaire S3: Human Papilloma Virus Knowledge and Acceptance Questions for Parents

For each of the following statements indicate your opinion on the scale ranging from 1 *(strongly disagree)*, 2 *(disagree)*, 3 *(neutral)*, 4 *(agree)*, and 5 *(strongly agree)* (adapted from Thomas, T. L., Strickland, O., Diclemente, R., & Higgins, M. [2013b]. *Parental human papillomavirus survey* [Database record]. Retrieved from PsycTESTS. http://dx.doi.org/10.1037/t24518-000)

1. HPV is a sexually transmitted disease.
2. Using condoms can prevent HPV.
3. Genital warts are caused by HPV.
4. People with HPV might not have symptoms.
5. HPV makes you unable to have children.
6. I worry that my child/ren might get HPV.
7. HPV can cause cervical cancer.
8. Treatment for HPV is painful.
9. Required vaccinations protect my child/ren from getting diseases from unvaccinated people.
10. I understand exactly for what the HPV vaccine is used.
11. Having genital warts makes it very difficult to find a sexual partner.
12. People should only get vaccinated for serious diseases.
13. I am more likely to trust vaccinations that have been around awhile.
14. Vaccinations are getting better all the time because of research.
15. Healthy children do not need vaccinations.
16. A vaccine against HPV could prevent future problems for my child/ren.
17. Giving my child/ren a new vaccine is like performing an experiment on my child/ren.

18. Most people I know think vaccinating children with the HPV vaccine before they are teenagers is a good idea.
19. A teenager should be able to get the HPV vaccination without a parent’s consent.
20. If this new HPV vaccine was available when I was an infant, I would be vaccinated against HPV infection.
21. Shots are very painful for my child/ren so I would rather my child/ren not get vaccinated.
22. If the new HPV vaccine is not required, my child/ren will not get vaccinated.
23. I think that even if the vaccine is expensive my child/ren will be able to get vaccinated.
24. Generally I do what my doctor recommends so I will get my child/ren vaccinated.
25. When I decide to get my child/ren vaccinated, I believe I will be able to get my child/ren vaccinated; in other words, I feel confident I can get my child/ren vaccinated.

For the next two questions indicate your opinion on the scale ranging from 1 *(very unlikely)*, 2 *(somewhat unlikely)*, 3 *(somewhat likely)*, and 4 *(very likely)* (adapted for young adults from Constantine, N. A. & Jerman, P. [2007b]. *HPV vaccination-related interview* [Database record]. Retrieved from PsycTESTS. http://dx.doi.org/10.1037/t09635-000

26. If an HPV vaccine were available, how likely would your child/ren have been vaccinated before their 13th birthday? (If the respondent answers question #26 with *very likely*, then question #27 is skipped, and the answer is assumed to be also *very likely)*.
27. If an HPV vaccine were available, how likely would your child/ren have been vaccinated before their 16th birthday? What are your reasons for feeling this way about vaccinating your child/ren? (English free text response limited to 50 words, qualitative question #1)

For each of the following questions select yes or no based on your opinion (adapted for young adults from Morales-Campos, D. Y., Markham, C. M., Peskin, M. F., & Fernandez, M. E. [2013].

Hispanic mothers' and high school girls' perceptions of cervical cancer, human papilloma virus, and the human papilloma virus vaccine [Appendix 1. Focus group guide questions]. *Journal of Adolescent Health, 52*(5), S69-75. http://dx.doi.org/10.1016/j.adohealth.2012.09.020

28. Do you think teen girls could get cervical cancer?
29. Have you heard of the human papillomavirus or HPV?
30. Do you think teenagers could get HPV?
31. Have you heard about the HPV vaccine?

For each of the following questions, please write in your opinion in response to the question asked. (English free text responses limited to 50 words for each response, qualitative questions 2 – 18).

32. What do you think of when you hear the word *cancer*?
33. What do you think of when you hear *cervical cancer*?
34. What do you think causes cervical cancer?
35. Who do you think gets cervical cancer?
36. What are the reasons you believe teen girls could (not) get cervical cancer?
37. If you have heard of the human papillomavirus or HPV, where did you hear about HPV?
38. If you have heard of the human papillomavirus or HPV, what have you heard about HPV?
39. Who do you think gets HPV?
40. What are the reasons you believe teenagers could (or could not) get HPV?

41. If you have heard about the HPV vaccine, what have you heard?
42. If you have heard about the HPV vaccine, where did you hear it?
43. What are your feelings about your child/ren getting the HPV vaccine?
44. What are the reasons you have these feelings about your child/ren getting the HPV vaccine?
45. What do you think the good things (benefits) would be of your child/ren getting the HPV vaccine?
46. What are your worries about your child/ren getting the HPV vaccine?
47. What might keep your child/ren from getting vaccinated?
48. How do you think the cost would affect your decision to have your child/ren vaccinated?

Survey item analysis categorization

Knowledge items: 1, 2, 3, 4, 5, 7, 8, 9, 10, 11, [12].

Acceptance items: 6, [13], 14, [15], 16, [17], 18, 19, 20, [21], [22], 23, 24, 25.

Post-ARP knowledge items: 29, 30, 31, 32, 33, 34, 35, 36, [37], 38, 39, 40, 41, 42, 43.

Post-ARP acceptance items: 26, 27, 28, 44, 45, 46, 47, 48, 49.

For question numbers in brackets a positive response is a negative association for the category.

A positive response indicates inadequate knowledge of non-acceptance of vaccination.

Permission S1: A. T. Still University Institutional Review Board Approval

Institutional Review Board
A. T. Still University
EXEMPT REQUEST APPROVAL

DATE: January 6, 2015

TO: Oroma Beatrice Afiong Nwanodi,

MD FROM: ATSU, Arizona IRB Committee

SUBJECT: Exemption from IRB Review, IRB Application #2014-237

Your application to the ATSU, Arizona IRB regarding "Multimodal Counseling Interventions' Effect on Human Papilloma Virus Vaccination Acceptance," proposes to collect no data associated directly or through identifiers linked to participants. The research will be conducted via on-line surveys collecting anonymous, de-identified data.

You may now proceed with data collection. If your project deviates from materials submitted in your application to the ATSU, Arizona IRB requesting exemption, you must seek additional approval or exemption from the ATSU, Arizona IRB prior to any work involving human participants being undertaken. Please forward to the IRB, a final copy of all study instruments posted on-line. Otherwise, no further reporting to the ATSU, Arizona IRB is required. Please keep this letter for your study files to verify IRB review.

If you have any questions, please feel free to call or

write. Sincerely,

D. _'/! '
-t-__.,; ..--j q-('·--n,.. !

Denise Lenik, CIP
Coordinator, ATSU, Arizona Institutional Review Board
AT. Still University
5850 East Still Circle
Mesa, AZ 85206
Main: (480) 219-6000
Office: (480) 245-6242
MesaiRB@atsu.edu

Cc: Helen Salisbury, PhD

Intervention S1: 14-sentence Control Counseling Piece

Cancer happens when the cells in the body grow out of control and become abnormal. If cancer is not treated, it can grow and spread to other parts of the body and can even cause death. Some cancers can be treated if they are found early or can even be prevented. Cancers are named after the part of the body where the abnormal cells are located. Cervical cancer is cancer of the neck of the uterus. The neck of the uterus is called the cervix. It is found in the lower part of the uterus. The human papillomavirus or HPV is passed through sexual contact. Although most HPV infections are temporary and people do not have any symptoms, some types of HPV infections cause abnormal changes in the cells of the cervix, which might lead to cervical cancer if not treated. These abnormal cell changes can be found by doctor using a test called a Pap test or Pap smear. Human papilloma virus can also affect men and in rare cases can cause cancer of the anus and penis.

A vaccine has been developed to prevent against certain types of HPV. The vaccine prevents boys, girls, men, and women from getting infected with certain types of HPV that cause cervical cancer. The HPV vaccine is recommended for boys, girls, men, and women 11 to 26 years old before they are sexually active. The reason for vaccinating girls and boys before they are sexually active is that HPV is a virus that is transmitted through sexual contact. The HPV vaccine will not treat or cure boys, girls, men, or women who already have been infected with the HPV virus. The HPV vaccine does not replace the need for a Pap test.

Adapted from Morales-Campos, D. Y., Markham, C. M., Peskin, M. F., & Fernandez, M. E. (2013). Hispanic mothers' and high school girls' perceptions of cervical cancer, human papilloma virus, and the human papilloma virus vaccine [Appendix 2. Focus group guide moderator descriptions]. *Journal of Adolescent Health, 52*(5), S69-75. http://dx.doi.org/10.1016/j.adohealth.2012.09.020

Intervention S2: Patient Education Human Papilloma Virus Handout

Public Health Fact Sheet. Patient information about HPV and the HPV vaccine

What is HPV? HPV (human papillomavirus) is a highly contagious virus that is spread by skin-to-skin contact. It can infect the skin, the mouth, the rectum and the anus. It can also infect male and female genital areas, including the skin on the penis or the vulva (area around the vagina), as well as the lining of the vagina and the cervix (the lower part of the uterus that is connected to the vagina). There are over 100 types of HPV, about 40 of which can be sexually transmitted.

What are the signs and symptoms of genital HPV infection? HPV can live on the skin or mucous membranes, including those in the genital tract, without causing any signs or symptoms. Most people do not know that they are infected with HPV, and most people infected with HPV will eventually clear the infection on their own without treatment. HPV can cause warts, or papillomas. Certain types of HPV (called *low-risk* types) cause common skin warts that often appear on the hands and feet. Other types of low-risk HPV can cause genital warts. Genital warts are single or multiple growths or bumps that often resemble a bumpy piece of cauliflower and can appear on the vulva, vagina, cervix, penis, scrotum, groin or thigh. These warts may appear weeks to months after sexual contact with a person who is infected.

Does HPV cause cancer? Yes, it can. Infection with some types of HPV, called *high-risk* HPV, does not cause warts but occasionally leads to cancer of the cervix. More rarely, these types of HPV can also lead to cancer of the vulva, vagina, anus or penis. Cervical cancer develops in about 1 in 152 (0.7%) American women in their lifetime or 12,000 American women each year. Cervical cancer causes 1 in 435 (0.2%) deaths of American women in their lifetime or 4,000 American women each year. This makes cervical cancer the 14th most common cause of cancer-related deaths in the United States (U.S.).

How is HPV transmitted? HPV is one of the most common sexually transmitted infections. As many as 75% of people who are sexually active will have at least one HPV infection in their lifetime. Genital HPV can be spread by sexual touching and by other types of sexual activity, such as intercourse. Pregnant women with genital HPV infection can pass HPV to their babies during vaginal delivery leading to HPV infection of the mouth, voice box or throat.

How are HPV infections diagnosed? Genital warts are diagnosed by looking at the genital area. An HPV infection of the cervix can be detected by a Papanicolaou (Pap) smear, which involves collecting cells from the cervix. These cells are checked in a laboratory for cancerous or precancerous changes caused by HPV infection. All types of HPV can cause mild changes in the cells of the cervix. In 90% of women, these changes become undetectable within 2 years. About 10 of the 40 genital HPV types can lead to changes in the cervix that may become cancerous.

How can the risk be reduced? Refraining from genital contact with another person or using a condom will reduce the risk of HPV infection. Condoms are not 100% effective because they only protect the covered area, and uncovered genital warts are infectious. Other forms of birth control, including diaphragms, birth control pills and IUDs, do not protect against HPV infection. Receiving a regular Pap smear is a very effective method of preventing disease caused by HPV infection. In 2006 and 2009 HPV vaccines were approved for females aged 9–26 in the U.S. In 2010 one HPV vaccine was also approved for males in the U.S. aged 9-26. In studies precancerous and cancerous changes of the cervix developed in 1% (86/8268) of unvaccinated females, and 0.01% (1/8301) of vaccinated females. Genital warts developed in about 3% (60/2278) of females who were unvaccinated, but in none who were vaccinated.

What is in the HPV vaccine? The HPV vaccine contains purified, inactive proteins that come from the 4 most common types of HPV: HPV types 6 and 11 (which cause 90% of genital warts)

and HPV types 16 and 18 (which cause 70% of cervical cancer). The vaccine does not protect against other types of HPV and does not contain any antibiotics or preservatives such as thimerosal.

How does the vaccine work? Three injections of the vaccine are required, at 0, 1 - 2 and 6 months. The vaccine is primarily effective in females who have not yet been exposed to HPV; thus, it is recommended that females be vaccinated before they become sexually active. The vaccine offers protection for at least 5.5 years, and studies are being performed to determine whether a booster dose will be required. Even with the vaccine, regular Pap smears are essential because the vaccine does not protect against all types of HPV that can cause cervical cancer.

Is the vaccine safe? Yes. The most common side effect is soreness around the injection site in the arm. Few other minor side effects were reported in the studies.

How much does it cost? In the U.S. the vaccine costs $390 - $450 for the 3 required doses, at about $130 - $150 per dose. HP V vaccination is covered by Medicaid and insurance purchased on health exchanges.

Originally written by: Tavé van Zyl Summer Intern, CMAJ., Eric Wooltorton MD MSc., Associate Editor, CMAJ., Noni MacDonald MD MSc Section Editor, Public Health, CMAJ

Competing interests: None declared.

Adapted and reproduced with permission from the *Canadian Medical Association Journal*, August 28, 2007, *177*(5) © 2007 Canadian Medical Association or its licensors.

Permission S2: Comments made on July 21, 2014 in the permission to adapt and reproduce the 14-sentence counseling brief as an intervention and use the focus interview question guide as survey questions

> "… I would advise you to keep the yes/no question paired with their follow-up questions. The reason for this is that if people respond "no" to an awareness question, there is no purpose in asking them knowledge questions about the topic because they know nothing about it. So these knowledge questions should be skipped. This would help alleviate respondent burden.
>
> I also wanted to make the disclaimer that my questions were developed for focus groups, so using open ended questions with an online survey format may not give you as rich data or could possibly cause respondent burden from participants having to write a detailed response to 17 open ended questions.
>
> With that being said, I wish you the best on your project. Thank you again for requesting permission to adapt/cite my work.
>
> Best regards,
>
> Daisy Morales-Campos, PhD"

Permission S3: Permission to use Risk Bites Video *Why vaccinate against HPV* from Dr. Maynard

Andrew Maynard <maynarda@umich.edu>

**To** :	o.nwanodi@juno.com
**Subject** :	Re: Permission to use Risk Bites video "Why vaccinate against HPV"
**Date** :	Thu, Jul 24, 2014 03:28 PM

"Hi Oroma,

Certainly - did you need a copy of the video, or can you work with the YouTube version?

Cheers

Andrew

Andrew D. Maynard
Chair, UM Department of Environmental Health Sciences
Director, University of Michigan Risk Science Center
NSF International Chair of Environmental Health Sciences
University of Michigan School of Public Health, 1415 Washington Heights, SPH Tower Room 6611
Ann Arbor, MI 48109
Tel: +1 734 647 6856, Email: maynarda@umich.edu

Web: http://riskcenter.umich.edu/2020-science

On Jul 24, 2014, at 5:15 PM, o.nwanodi@juno.com wrote:

Dr. Maynard:

I am a candidate for the Doctor of Health Science at A. T. Still University. For my applied research project, I would like to evaluate the effect of counseling interventions, including videos such as "Why vaccinate against HPV" on opinions about HPV and HPV vaccines. Therefore, I would appreciate permission to use your video "Why vaccinate against HPV" as part of a SurveyMonkey Audience online survey.

Your consideration is appreciated.

Best regards,

Oroma Nwanodi, MD, MS-CROM, FACOG, ABIHM"

Permission S4: Consent to Adapt and Reproduce the Public Health Fact Sheet

1/14/2015 Access Copyright – RMS

https://portal.accesscopyright.ca/Portal/TXLicence/PrintLicence.aspx?licence_id=109651&invoice_id=46147&lctype=TX 1/5

Title	Patient information about HPV and the HPV vaccine
Article	Yes
Medium	Digital
Use type	Presentation
Pages	462
No. of pages	1
Licensed users	1600
Total copies	1600
Amount	$300.00 Subtotal $300.00 Admin fee $15.00 Total $315.00

This is a Transactional Licence purchased online

Licensee:	Oroma Nwanodi
Licence Number:	TX00109651 On-line
Payment:	Yes
Start Date:	01/14/2015 Request

Date: 01/14/2015

Comments: For permission to adapt the following and post to a password

protected site for 1600 users only

Work Information Invoice Information:

Invoice Number: 46147

Invoice Date: 01/14/2015 Sub total: $300.00

Admin fee: $15.00

Total: $315.00

ONLINE TRANSACTIONAL LICENCE AGREEMENT

INTRODUCTION

THIS AGREEMENT SETS OUT THE TERMS AND CONDITIONS OF THE LICENSEE'S (you, your) PERMITTED COPYING AND USE OF COPYRIGHT-PROTECTED MATERIALS, IN PRINT OR DIGITAL FORM. THE AGREEMENT COMPRISES THIS DOCUMENT AND THE ORDER DETAILS, WHICH IDENTIFIES THE MATERIALS FOR WHICH PERMISSION HAS BEEN SOUGHT. THIS AGREEMENT IS AN IMPORTANT LEGAL DOCUMENT AND YOU SHOULD READ IT CAREFULLY. UPON CLICKING "SUBMIT ORDER", A BINDING LEGAL CONTRACT WILL EXIST BETWEEN YOU AND THE CANADIAN COPYRIGHT LICENSING AGENCY. IF YOU CLICK "SUBMIT ORDER" WITHOUT READING THIS AGREEMENT, YOU ARE STILL AGREEING TO BE

BOUND BY ALL THE TERMS AND CONDITIONS OF THIS AGREEMENT.

THE CANADIAN COPYRIGHT LICENSING AGENCY ENCOURAGES YOU TO PRINT A COPY OF THIS AGREEMENT AND RETAIN IT IN YOUR FILES.

2. GRANT OF LICENCE

This Agreement is between you and The Canadian Copyright Licensing Agency ("Access Copyright"). Access Copyright hereby grants you permission, on a non-exclusive basis, to copy and use the copyright-protected materials (the "Work(s)") identified on the Order Details, Licence Information and Work Information pages, in the 1/14/2015 Access Copyright - RMS

https://portal.accesscopyright.ca/Portal/TXLicence/PrintLicence.aspx?licence_id=109651&invoice_id=46147&lctype=TX 2/5 manners permitted (the "Permitted Uses"), by the number of users identified (the "Licensed Users"), in the quantities specified (the "Total Copies"), and for payment (the "Amount"), as set out in the Order Details, Licence Information and Work Information pages.

3. PERMITTED USES

The scope of the Permitted Uses of the Work(s) specified by the "Medium" and "Use Type" are identified in the Order Details, Licence Information and Work Information pages. Please note that licences for Digital Permitted Uses will expire when the set of students for which the licence was obtained completes or leaves the course. A new licence will need to be purchased for a new set of students/users:

(a) "Print” & “Reproduction”: means any reproduction of the Work(s) by photocopying, xerography, facsimile transmission and any other reprographic process. The Amount is based on the Total Copies, that is, the number of pages you copy multiplied by the number of Licensed Users specified, multiplied by the royalty fee. You may not produce more sets than a number equal to the number of Licensed Users first specified.

(b) "Print" & “Presentation”: means photocopying, xerography, transcription or drying onto acetate or other material for an overhead or slide projection and any other similar reproduction process. The Amount is based on the making of one print copy of the Work(s) for the overhead or slide projection, multiplied by the number of people attending or registered for the presentation, multiplied by the royalty fee. The number of Licensed Users shall not

exceed the number of Licensed Users first specified. You are entitled to make one print copy for each person in attendance or registered for the presentation

(c) “Print" & "Alternate Format”: means a Braille or large print (by a reprographic process) reproduction of all or part of the Work(s) produced for a person with a disability that inhibits that person from reading or viewing a work. The Amount is based on a flat fee for the reproduction of the entire Work(s), multiplied by the number of Licensed Users. The number of reproductions made shall not exceed the number of Licensed Users first specified.

(d) "Digital" & “Reproduction”: means the storing, downloading or e-mailing the Work(s) which you have converted into a digital format or which was already in digital format on any digital recording medium such as floppy disk, CD-ROM or personal computer. The Amount is based on the number of pages you copy, multiplied by the number of Licensed Users, multiplied by the

royalty fee. The number of people having access either by e-mail or by direct access to the digital recording medium shall not exceed that number of Licensed Users first specified.

(e) “Digital" & "Presentation”: means any digital reproduction of the Work(s) for the purpose of inclusion in any electronic presentation. The Amount is based on the making of one digital copy for inclusion in your electronic presentation, multiplied by the number of people attending or registered for the presentation, multiplied by the royalty fee. The number of Licensed Users shall not exceed the number of Licensed Users first specified. You are entitled to make one digital reproduction only and one print copy for each person in attendance or registered for the presentation

(f) "Digital" & “Alternate Format”: means an audio, electronic encoded or machine readable reproduction of all or part of the Work(s) produced for a person with a disability that inhibits that person from reading or viewing a work. The fee is based on a flat fee for the reproduction of the entire Work(s), multiplied by the number of Licensed Users. The number of digital reproductions made shall not exceed the number of Licensed Users first specified. You are also entitled to make one paper copy for each Licensed User

(g) “Digital” & "Intranet": means the posting of the Work(s) on a secure network to which electronic access is restricted. The fee is based on the number of pages you copy multiplied by the number of Licensed Users, multiplied by the royalty fee. The number of people having access to the secure network shall not exceed the number of Licensed Users first specified.

(h) “Digital” & "Internet": means the posting of the Work(s) on an open web site. The fee is based on the number of pages you copy and the duration of the time the work will be available.

4. LIMITATIONS

(a) The following limitations strictly apply to all Permitted Uses of the Work(s):

i. Only Licensed Users shall be provided with copies of the Work(s) under this Agreement.

ii. All reproductions, whether in print or digital form, will be accurate and faithful reproductions of the Work(s).

No modifications to the Work(s) are permitted and no comments or additional content may be incorporated into the Work(s).

iii. Other than as specifically permitted under this Agreement, any copying, use, distribution or further distribution, publication, republication, sale, communication to the public, or licensing of the Work(s), whether in digital, paper, or any other form, will result in this licence being automatically revoked and shall be deemed to be an infringement of copyright, rendering you liable to Access Copyright and the copyright owner(s) for all remedies arising from such infringement. For clarity, where a digital licence is purchased, Licensed Users are also entitled to a print copy (e.g. print a copy from a secure intranet).

iv. Permitted Uses are only for the Work(s), a copy of which you have lawfully obtained. You are not permitted to make print or digital reproductions of the Work(s) if it was obtained through

the circumvention of technical measures of protection or through a password-protected website and further copying is prohibited by the licence providing access.

v. You are not permitted to convert the Work(s) from a print format to a digital format where the work is commercially available in digital format.

vi. In the case of Intranet use of a Work(s), the Work(s) will be made available only within a network where electronic access is by means of electronic password, or other control, permitting access only to Licensed Users. At the request of Access Copyright, you shall provide us with the electronic password or other controlled access to the intranet to enable us to audit the integrity and security of the work. Your failure to do so within 15 calendar days of our request shall be a material breach of this Agreement.

vii. For intranet or internet use of a Work(s), the Work(s) shall be available only for the period of time for which you have been invoiced, where the time period specified on the licence confirmation as Use Period is different than that specified in the comments field, the comments field takes precedence.

viii. This Agreement cannot be combined with other Transactional Licence Agreements or with other Licence Agreements offered by Access Copyright, so as to permit any systematic or cumulative copying of the Work(s).

(b) You will include a prominent copyright notice on each print copy of the Work(s), or on the cover page of each set of print copies, or on all electronic copies of the Work(s). The notice will contain each of the following:

i. © symbol;

ii. name of copyright owner(s);

iii. name of author(s) if different from the copyright owner(s);

iv. year of first publication; and

v. a statement which reads: "This work is protected by copyright and the making of this copy was with the permission of Access Copyright. Any alteration of its content or further copying in any form whatsoever is strictly prohibited, except as otherwise permitted by law."

5. PAYMENT

While you may carry out all uses permitted by this Agreement immediately upon the start date set out in the confirmation, the licence is automatically revoked and is null and void as if it had never been given without further notice to you if complete payment including the administrative fee and taxes is not received by Access Copyright immediately when made through a payment agent such as a credit card company, or when made directly by you, within 30 days after an invoice has been issued by Access Copyright. If the licence is revoked, all indemnity provisions contained in this agreement will not apply to any use you may have made. There is a minimum payment of five dollars ($5.00), as well as an administrative fee of five percent (5%) on payments over five dollars ($5.00), all

exclusive of taxes.

6. INDEMNIFICATION

(a) Subject to clause 7(b) below, Access Copyright agrees to hold you harmless and to indemnify you for any monetary amounts which you are required to pay to another person relating to a claim arising solely as a result of your copying and use of the Work(s), provided that:

i. you provide written notice to Access Copyright of any claim within five business days of your learning of the claim; and

ii. you are not, at the time you provide written notice to Access Copyright, in breach of any term or condition in this Agreement.

(b) The indemnification in clause 6 excludes any claim which alleges a breach of any kind of the moral rights of an author.

7. TERM AND TERMINATION

(a) The licence granted to you is on a "one-time only" basis for Licensed Users.

(b) Your failure to pay any amount when due, or your use of the Work(s) beyond or outside of the scope of this Agreement, shall be a material breach of this Agreement, and shall cause this Agreement to terminate automatically without any further notice from Access Copyright.

(c) Upon expiry or termination of this Agreement, you will immediately delete all digital copies of the Work(s) from storage or your web site, and you will make no new uses of the Work(s) except by separate licence.

(d) All your obligations and those of Access Copyright which expressly, or by their nature, impliedly survive termination or expiry, shall continue in full force and effect subsequent to and notwithstanding such termination or expiry.

8. MISCELLANEOUS

(a) No variation to this Agreement is permitted. This Agreement is not transferable or assignable by you unless you obtain Access Copyright's written permission.

(b) The provisions of this Agreement are severable, and if any provision is found to be invalid or unenforceable, the remaining provisions of this Agreement shall not be affected thereby and shall continue in full force and effect.

(c) This Agreement, including the identifying information in the Order Details, Licence Information and Work Information pages constitute the entire agreement and understanding of you and Access Copyright and supersede any and all prior understanding or agreements regarding the subject matter of this Agreement.

(d) This Agreement will be governed by and interpreted in accordance with the relevant laws of the province of Ontario and Canada, and treated as a contract both signed and wholly performed in Ontario. The parties consent to the exclusive jurisdiction and venue of the Ontario Superior Court at Toronto in all disputes arising out of or relating 1/14/2015 Access Copyright - RMS

https://portal.accesscopyright.ca/Portal/TXLicence/PrintLicence.aspx?licence_id=109651&invoice_id=46147&lctype=TX 5/5 to the enforcement or alleged breach of this Agreement, without

prejudice to the right of Access Copyright and copyright owner(s) to advance any claim for copyright infringement in the Federal Court of Canada.

(e) The parties have required that this Agreement be drawn up in English. Les parties ont demandé que cette convention soit rédigée en anglais.
